# Selected Behavioural Factors Affecting Oral Health in Schoolchildren: Results from the Health Behaviour in School-Aged Children (HBSC) Slovak Study

**DOI:** 10.3390/ijerph17207516

**Published:** 2020-10-15

**Authors:** Eliška Štefanová, Tibor Baška, Jana Holubčíková, Silvia Timková, Mária Tatarková, Miroslava Sovičová, Henrieta Hudečková

**Affiliations:** 1 Department of Public Health, Jessenius Faculty of Medicine in Martin, Comenius University in Bratislava, Malá Hora 11149/4B, 036 01 Martin, Slovakia; eliska.stefanova@gmail.com (E.Š.); tibor.baska@jfmed.uniba.sk (T.B.); tatarkova12@uniba.sk (M.T.); duranova31@uniba.sk (M.S.); henrieta.hudeckova@jfmed.uniba.sk (H.H.); 2 Department of Health Psychology, Faculty of Medicine, Pavol Jozef Šafárik University in Košice, 040 11 Košice, Slovakia; janka.holubcikova@gmail.com; 3 1st Department of Stomatology, Faculty of Medicine, Pavol Jozef Šafárik University in Košice and University Hospital of Luis Pasteur, 040 11 Košice, Slovakia

**Keywords:** behavioural factors, dental caries, toothbrushing, oral health, sugar consumption, adolescents, Health Behaviour in School-Aged Children

## Abstract

Oral diseases, particularly dental caries, affect as much as nine in 10 persons globally. Its development starts during childhood. Behavioural factors play an important role in its aetiology. The aim of the research was to analyse the prevalence of selected behavioural factors associated with dental caries in Slovak adolescents. Selected factors, such as toothbrushing less than once a day, consumption of sweets and sweetened soft drinks daily and their combination, were analysed using data from Health Behaviour in School-Aged Children surveys carried out in 2005/2006, 2009/2010, 2013/2014 and 2017/2018 in Slovakia. The target group consisted of 11- to 13-year-old schoolchildren. The results were analysed by sex and socioeconomic status. The consumption of sweets and sweetened soft drinks, despite declining, remains widespread (41.3% of boys and 39.6% of girls in 2017/2018). The absence of daily toothbrushing, similarly as a co-occurrence of factors, were more frequent in boys (10.6% and 5.0% in 2017/2018, respectively) than in girls (5.1% and 2.3% in 2017/2018, respectively). The absence of daily toothbrushing was associated with a lower socioeconomic situation. In conclusion, behavioural risk factors affecting oral health are widespread in Slovak adolescents. Despite the positive development of the epidemiological situation, effective interventions, as well as the improvement of oral hygiene in lower socioeconomic groups, are needed.

## 1. Introduction

Oral health is defined as a normal state of the oral cavity, with the individual’s ability to eat, smile, speak, etc. without pain of any kind or noticeable disease [1]. It plays an important role in maintaining overall health [2]. Dental caries ranks among the most common oral diseases. Due to its prevalence, economic aspect and effect on the quality of life, it presents a significant public health issue [3,4,5,6].

There are numerous factors increasing a risk of dental caries. Beside inherent and metabolic predispositions, behavioural factors are of great importance. Among them, oral hygiene and diet play significant roles [1,7,8]. Tooth-brushing applied at least once a day has been considered as a principal tool to maintain oral health and to prevent caries [9]. The absence of toothbrushing is associated with 2.1 times higher prevalence of developing dental caries in comparison to regular toothbrushing [10]. Results from Spain showed a higher prevalence of dental caries in the group of 12-year-old schoolchildren brushing their teeth once a day or not at all [11]. Diets rich in the consumption of sweetened foods, sweetened soft drinks and energy drinks promote the initiation and further development of dental caries [12]. A significant improvement was detected after cutting the sugar intake to less than 5% of the all-day intake [13].

The age of 12 is generally considered as a crucial period for the development of dental caries and further impaired oral health. It is mostly because, in the majority of children, all the permanent teeth, except the third molars, have already erupted [14]. The average DMFT (Decayed, Missing and Filled Teeth) index in 12-year-old children in Slovakia is 1.71, which is higher than the European average DMFT index [14,15].

Differences in behaviours connected with caries are detected with respect to sex, e.g., girls are, in general, more interested in their oral health and brush their teeth more regularly, but they also perceive the consumption of sweetened food as more excessive in comparison to boys [16,17,18]. 

The socioeconomic situation can be considered as an independent determinant of tooth decay. Significantly, more cases of dental caries are present in children who grow up in lower socioeconomic families, in combination with a low income and low education level [19]. It seems that the situation is caused by differences in oral hygiene and dietary habits. Higher soft drinks consumption was detected in children from low socioeconomic families who brushed their teeth sporadically [20,21,22]. On the other hand, good oral hygiene is associated with a higher education level, mostly in mothers, and a higher socioeconomic level corresponds with the probability of toothbrushing twice a day or more [23,24,25].

The research focused on the prevalence of behavioural factors of dental caries, namely indicators of insufficient dental hygiene (toothbrushing less than once a day) and eating habits associated with an increased risk of caries (eating sweets daily, drinking soft drinks daily and their combination) among 11- to 13-year-old children. The aim was to fill the missing gap in knowledge relating to epidemiological characteristics of the behaviours responsible for dental caries. The data available from dentists deal mostly with the microbiological origin and clinical aspects of dental caries. Moreover, data provided by dentists cannot be considered as population-based ones, since there is a significant proportion of children not attending regular check-ups and treatments [7,14,26]. 

The analysed data originated from Health Behaviour in School-Aged Children (HBSC) surveys carried out in Slovakia in school years 2005/2006, 2009/2010, 2013/2014 and 2017/2018. HBSC surveys provide base valid representative epidemiological data. Behavioural factors of dental caries of Slovak children are analysed by sex and socioeconomic status, with identified trends over time. 

The research analyses the prevalence of insufficient toothbrushing (less than once a day), eating of sweets and/or drinking of sweetened soft drinks and co-occurrence of the two above-mentioned factors in relation to sex and socioeconomic status. Understanding the epidemiology of risk behaviours associated with dental caries during childhood, as well as defining the subpopulations in increased risks, can considerably help to design and implement effective preventive intervention programs tailored for this target population. The results of the research, along with providing the estimated prevalence of the children population at risk considering basic sociodemographic determinants, can contribute to better understanding the issue as such. 

## 2. Materials and Methods 

### 2.1. Design of the Study 

Health Behaviour in School-Aged Children (HBSC) is an international, school-based cross-sectional survey carried out every 4 years. The HBSC study focuses on health behaviour in 11-, 13- and 15-year-old schoolchildren. Its standardised design enables to create harmonised datasets appropriate for cross-country comparisons, as well as for identifying changes over time [26]. This research is based on the HBSC study and analyses four of the HBSC surveys carried out in Slovakia in school years 2005/2006, 2009/2010, 2013/2014 and 2017/2018. The design of the presented research can be characterised as a series of cross-sectional studies.

A study in school year 2005/2006 was carried out through the regional offices of public health in Slovakia. Since school year 2009/2010, the Department of Health Psychology of P. J. Safarik University has been the main coordinator of the HBSC survey. The study was approved by the Ethics Committee of the Medical Faculty at P. J. Safarik University in Kosice no. 82/2009 (2009/2010), no. EC09/2012 (2013/2014) and no. 16N/2017 (2017/2018). 

### 2.2. Sample

Two-step sampling was used, keeping the standardised research protocol. In the first step, participating schools were randomly selected with probability proportional to size using an official list of all schools obtained from the Slovak Institute of Information and Prognosis for Education. The sample of schools was stratified by region (eight administrative self-governing regions) and type of school (elementary schools comprising the 1st–9th grades and eight-year grammar schools comprising the 6th–13th grades). There were 87 involved schools in the year 2005/2006, 106 schools in 2009/2010, 130 schools in 2013/2014 and 109 schools in 2017/2018 (Table 1). 

In the second step, within the participating schools, classes were randomly selected to collect data. Schoolchildren from the 5th to 9th grades were considered as eligible, i.e., associated with 11- to 15-year-old adolescents. The analysis in this research included only 11- to 13-year-old respondents. The number of respondents in the last data collection—2017/2018—more than doubled in comparison to 2005/2006. The ratio of boys and girls in the sample was approximately the same (Table 1). 

Parents were informed in advance about the study via school administration, and using a written informed consent form, they could opt out if they disagreed with their child’s participation. Participation was fully voluntary and anonymous, with no explicit incentives provided for participation. 

### 2.3. Questionnaire

To collect data, uniform anonymous questionnaires were filled out in classes at schools by schoolchildren. Validated standard questionnaire for the HBSC study contains a wide range of questions focused on health behaviours in schoolchildren, such as eating habits; oral health; risk behaviours (alcohol, tobacco, cannabis, etc.); peers; physical activity; sexual health; etc. [27]. This research analysed only select questions from the HBSC questionnaire (see the Section 2.5 *Analysed Variables*).

A supervisor was present to help the children if needed and to ensure the credibility of the study. Questionnaires were paper-based, except for the school year 2017/2018, when an online version of the questionnaire was launched. 

### 2.4. Data Collection

To make the HBSC cross-sectional survey eligible, it was necessary to collect data from around 1500 respondents in each age group each year [27]. Data were collected approximately in a period from April to June 2006, 2010, 2014 and 2018 (the period of months varied between the years) through trained supervisors. A team for data collection underwent partial personal changes throughout the years, but the same standard approach in their training was used. 

### 2.5. Analysed Variables

Most of the questions had several options to be answered to offer a wider range to express the respondents’ views. However, dichotomisation was used to clearly analyse the respective variables.

Toothbrushing was measured by the question “How often do you brush your teeth?” Possible responses were “More than once a day”, “Once a day”, “At least once a week but not daily”, “Less than once a week” and “Never”. After dichotomisation, the proportion of answers “At least once a week but not daily”, “Less than once a week” and “Never” was analysed as a risk behaviour.

The consumption of sweets in schoolchildren was measured by the question “How many times a week do you usually eat sweets (candy or chocolate)?” Possible answers were “Never”, “Less than once a week”, “Once a week”, “2–4 days a week”, “5 to 6 days a week”, “Once a day every day” and “Every day, more than once”. After dichotomisation, the proportion of answers “Once a day every day” and “Every day, more than once” was analysed as a risk behaviour.

The consumption of sweetened soft drinks was measured by the question “How many times a week do you usually drink coke or other soft drinks that contain sugar?” Possible answers were “Never”, “Less than once a week”, “Once a week”, “2–4 days a week”, “5 to 6 days a week”, “Once a day every day” and “Every day, more than once”. The proportion of answers “Once a day every day” and “Every day, more than once” was analysed as a risk behaviour.

The socioeconomic status (SES) of a family is, in the HBSC research, defined by the family affluence scale (FAS), which consists of four questions: “Does your family own a car, van or truck?” (No = 0, Yes, one = 1 and Yes, two or more = 2); “Do you have your own bedroom for yourself?” (No = 0 and Yes = 1); “How many computers does your family own?” (None = 0, One = 1, Two = 2 and More than two = 3) and “How many times did you and your family travel out of Slovakia for a holiday/vacation last year?” (Not at all = 0, Once = 1, Twice = 2 and More than twice = 3). In the 2014 HBSC data collection, the FAS was updated, and two more questions were added to this instrument: “How many bathrooms (room with a bath/shower or both) are in your home?” (None = 0, One = 1, Two = 2 and More than two = 3), and “Does your family have a dishwasher at home?” (No = 0 and Yes = 1). The final score of every respondent determined the SES. Values of FAS up to the median were considered as a lower SES subpopulation and above the median as a higher one.

### 2.6. Statistical Analysis 

The results are expressed as a percentage (%) with the respective 95% confidence intervals. Differences were statistically evaluated using a chi-square test. As a level of statistical significance, *p* < 0.05 was considered. To test changes across time, Bonferroni correction was applied for post-hoc pairwise comparisons.

## 3. Results

Within the analysed factors, the daily consumption of sweets and/or sweetened soft drinks was widespread, affecting a considerable proportion of children (ranking from 57.9% of boys in 2005/2006 to 39.6% of girls in 2017/2018). The absence of daily toothbrushing uniformly dominated in boys through the studied period and varied from 10.6% (boys in 2017/2018) to 3.6% (girls in 2013/2014). The dominance of boys was projected also in a co-occurrence of insufficient toothbrushing with the consumption of sweets and/or sweetened soft drinks, and its prevalence was highest in boys in 2005/2006 (7.1%) (Table 2). 

A higher prevalence of insufficient toothbrushing was strongly associated with a lower socioeconomic situation in both boys and girls (Table 3 and Table 4). The daily consumption of sweets or sweetened soft drinks differed only marginally across the socioeconomic groups. A significant difference was present in girls in 2013/2014 (47.7% vs. 42.1%), with a higher consumption in low FAS groups. Insufficient toothbrushing combined with the consumption of sweets or sweetened soft drinks dominated among respondents from lower socioeconomic groups only in girls in 2005/2006 and both in boys and girls in 2017/2018 (Table 3 and Table 4).

**Figure 1:**
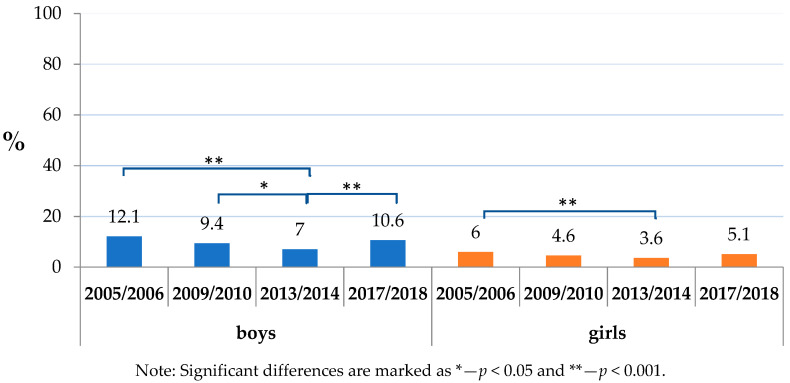
Toothbrushing less than once a day by sex.

Insufficient toothbrushing prevalence was highest in 2005/2006, including 12.1% of boys and 6.0% of girls (Figure 1). It gradually declined until 2013/2014 both in boys and girls, reaching the lowest rates (7.0% and 3.6%, respectively). However, the declining trend no longer continued in 2017/2018. It even significantly increased in boys up to 10.6%.

**Figure 2:**
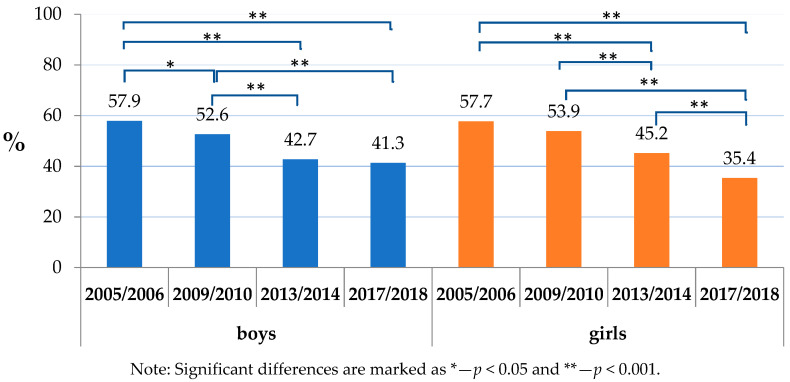
Consumption of sweets or sweetened soft drinks by sex.

Almost six in 10 respondents reported in 2005/2006 the daily consumption of either sweets or sweetened soft drinks (Figure 2). A noticeable declining trend was shown both in boys and girls, leading to 41.3% and 35.4% in 2017/2018, respectively.

**Figure 3:**
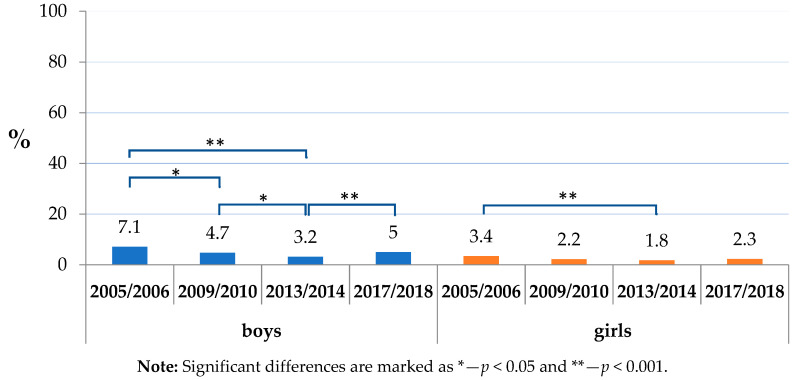
Toothbrushing less than once a day and the consumption of sweets or sweetened soft drinks by sex.

The co-occurrence of the above-mentioned factors, i.e., insufficient toothbrushing together with the daily consumption of sweets or sweetened soft drinks, was seen in less than one-tenth of the respondents (Figure 3). It showed a declining trend until 2013/2014. In 2017/2018, a repeated increase was observed, reaching statistical significance only in boys (from 1.8% to 2.3%).

## 4. Discussion

As the results indicate, the daily consumption of sugar, despite a declining trend, has remained among the most prevalent factors associated with dental caries, affecting the oral health of more than one-third of schoolchildren aged 11–13 years. This holds both for boys and girls, as well as all socioeconomic groups. Worth mentioning is a decrease between 2005/2006 and 2017/2018. For boys, it was from 57.9% (2005/2006) to less than a half (41.3% in 2017/2018). In girls, it was from 57.7% in 2005/2006 to 35.4% in 2017/2018. Another study proved similar findings, i.e., a high consumption of sweets in schoolchildren, whereas a frequent consumption of sweets or sweet beverages was associated with a higher risk of dental caries [28].

Insufficient toothbrushing is less common. However, a positive trend seen in 2013/2014 broke up, and according to the last survey carried out in 2017/2018, the situation has returned to be similar to 2005/2006. As the results implicate, this is mostly the issue of boys and lower socioeconomic groups of the population. Moreover, about half of those not brushing their teeth also consume sugars on a daily base. Such a combination presents a particularly high risk for dental caries [29]. Although present in less than 5% of the respondents, it should not be under-evaluated. Taking into consideration the official statistical population data (162,530 children in age of 11–13 years old up to the date of June 30, 2018 in Slovakia), this problem relates to as much as about 8000 children of this age group in the country [30]. Moreover, it is important to keep in mind that the analysed data are subjective; thus, underreporting can potentially present “a tip of the iceberg” effect, making the problem even deeper [31]. 

As already mentioned, the insufficient toothbrushing relates particularly to boys. One of the possible causes may be that girls, or women in general, consider oral health as important, with a positive impact on their quality of life. Good oral health represents a positive attitude towards their own appearance. It could be also related to the different perception of health as such [32,33]. Girls in general tend to consume more sweets and/or sweetened soft drinks due to a need of stress and depression reduction [34]. On the other hand, their toothbrushing practices are better than in boys. The study shows that the daily consumption of sweets linked with toothbrushing more often results in a lower risk of dental caries [28]. 

Another problem to be considered is the quality of toothbrushing. As many studies showed, the problem is mostly in the wrong technique (incorrect brushing movements, insufficient time of brushing, etc.), which may be, in the long term, harmful to oral health. Although, this problem is, in many cases, preventable through proper education [35,36,37,38,39]. Therefore, it is necessary to teach children not only to brush their teeth but to brush them properly. This research only analysed whether or not they brush their teeth. For future research, it could be interesting to deeply analyse the determinants of toothbrushing and the quality of toothbrushing techniques. 

The results showed an association in relation to the socioeconomic situation. The main problem was detected in families where the parents’ educational level was low. Parents with lower educational levels do not place an importance on toothbrushing, probably due to a lack of information, i.e., low health literacy [5,23]. A lack of information in parents is associated with a low level of motivation for shaping children’s oral habits [40]. However, it is necessary for parents to be a motivational factor in the subject of oral hygiene [33,41]. Parents, especially mothers, play a very influential role in creating children’s habits [33,40]. The challenge for public health is to better focus educational and information activities on families with lower socioeconomic positions to improve behavioural factors in children.

### Limitations and Strengths

One of the obvious limitations is the response rate. Dropouts were caused mostly by the absence of children due to illness or other personal reasons. In the data collection of 2017/2018, there were some technical difficulties (due to the online version of the questionnaire) possibly affecting the final number of participants. However, the assumption is that these reasons were not related to the analysed issue and did not significantly bias the results. Moreover, comparable response rates were achieved also in other similar studies [42].

Another potential limitation of the research is that the HBSC data did not provide a comprehensive picture on a risk but only some aspects of it. For example, taking into consideration the data on oral health—namely, the caries index—in the studied population could contribute to a more complex understanding of the issue. Therefore, findings should be considered rather as an insight into the epidemiological situation and its trends and changes over time. These pieces of information, despite their limited scope, provide an important groundwork for population-based preventive measures, as well as relevant projections of the situation in the future. The strongest point of the analysis was the use of representative data, including the whole target population of the given age group and the analysis of dental caries and its association with eating habits, supplemented by different combinations of eating habits. Most of the studies dealing with oral health employ data from dentists [14,43,44]. According to the HBSC Slovakia report, as much as 15% of boys and 12% of girls aged 13 years reported not visiting the dentist during recent years [45]. Subjective data are limited but still valid. Moreover, as the latest official data on dental care show, among children and adolescents (six to 14 years old) as much as one-quarter of them have not been registered for dental care [15,29], i.e., objective data coming from dentists are limited to a population attending check-ups and undergoing dental care and can overlook a considerable proportion of the population. Therefore, research findings fulfil this information gap. 

## 5. Conclusions

Eventually, despite a decline of the daily eating of sugar within recent years, it remains a widespread factor associated with a poor quality of oral health in children. Its combination with insufficient toothbrushing is mostly a problem for boys and lower socioeconomic population groups, where a particularly high risk can be expected. There is a need to find out effective ways for how to address these target groups in preventive programs, with an emphasis on youngsters when habits are still developing.

## Figures and Tables

**Table 1:** Characteristics of the Health Behaviour in School-Aged Children (HBSC) survey samples carried out in school years 2005/2006, 2009/2010, 2013/2014 and 2017/2018.

School Year	Number of Schools Involved in the Survey	Total Number of Respondents	Number of 11 to 13-Year-Old Respondents (Boys; Girls)	Response Rate (%)
2005/2006	87	3877	2525 (1203; 1322)	86
2009/2010	106	4308	2740 (1302; 1438)	80
2013/2014	130	5245	3696 (1811; 1885)	79
2017/2018	109	8902	5260 (2651; 2609)	60

**Table 2:** Prevalence of the selected behavioural risk factors of dental caries in 11–13 year-old children by sex.

School Year	Sex	Absence of Daily Toothbrushing		Daily Consumption of Sweets and/or Sweetened Soft Drinks		Co-occurring of Daily Toothbrushing Absence with Consumption of Sweets and/or Sweetened Soft Drinks	
		Absolute Number (Abs.) (%)	Difference (Diff.) (*p*-Value)	Abs. (%)	Diff. (*p*-Value)	Abs. (%)	Diff. (*p*-Value)
2005/2006	Boys	145 (12.1)	**<0.001**	696 (57.9)	0.951	85 (7.1)	**<0.001**
	Girls	86 (6.0)		821 (57.7)		49 (3.4)	
2009/2010	Boys	203 (9.4)	**<0.001**	1141 (52.6)	0.399	102 (4.7)	**<0.001**
	Girls	108 (4.6)		1278 (53.9)		53 (2.2)	
2013/2014	Boys	187 (7.0)	**<0.001**	1142 (42.7)	0.071	85 (3.2)	**<0.001**
	Girls	98 (3.6)		1229 (45.2)		49 (1.8)	
2017/2018	Boys	280 (10.6)	**<0.001**	1096 (41.3)	0.196	133 (5.0)	**<0.001**
	Girls	132 (5.1)		1033 (39.6)		61 (2.3)	

Note: Abs.—absolute number and Diff.—difference; Bold font indicates the presence of statistical significance.

**Table 3:** Prevalence of the selected risk factors of caries by socioeconomic status in 11-13-year-old boys.

School Year	FAS	Absence of Daily Toothbrushing		Daily Consumption of Sweets and/or Sweetened Soft Drinks		Co-occurring of Daily Toothbrushing Absence with Consumption of Sweets and/or Sweetened Soft Drinks	
		Abs. (%)	Diff. (*p*-Value)	Abs. (%)	Diff. (*p*-Value)	Abs. (%)	Diff. (*p*-Value)
2005/2006	Low FAS	85 (13.9)	**0.034**	365 (59.8)	0.265	49 (8.0)	0.130
	High FAS	48 (9.8)		278 (56.5)		28 (5.7)	
2009/2010	Low FAS	105 (10.6)	**0.028**	507 (51.0)	0.113	52 (5.2)	0.161
	High FAS	67 (7.6)		480 (54.7)		34 (3.9)	
2013/2014	Low FAS	95 (7.9)	**0.008**	500 (41.7)	0.556	45 (3.8)	0.053
	High FAS	55 (5.2)		458 (42.9)		25 (2.3)	
2017/2018	Low FAS	128 (11.7)	**0.008**	452 (41.3)	0.540	66 (6.0)	**0.002**
	High FAS	60 (7.9)		302 (39.9)		22 (2.9)	

Note: FAS—family affluence scale, Abs.—absolute number and Diff.—difference; Bold font indicates the presence of statistical significance.

**Table 4:** Prevalence of the selected risk factors of caries by socioeconomic status in 11-13-year-old girls.

School Year	FAS	Absence of Daily Toothbrushing		Daily Consumption of Sweets or Sweetened Soft Drinks		Absence of Toothbrushing Combined with Daily Consumption of Sweets or Sweetened Soft Drinks	
		Abs. (%)	Diff. (*p*-Value)	Abs. (%)	Diff. (*p*-Value)	Abs. (%)	Diff. (*p*-Value)
**2005/2006**	Low FAS	60 (7.0)	**0.007**	507 (58.7)	0.223	36 (4.2)	**0.015**
	High FAS	16 (3.4)		261 (55.3)		8 (1.7)	
2009/2010	Low FAS	74 (5.8)	**0.002**	689 (53.7)	0.808	37 (2.9)	0.054
	High FAS	25 (2.9)		474 (54.2)		14 (1.6)	
2013/2014	Low FAS	58 (4.3)	**0.014**	644 (47.7)	**0.006**	28 (2.1)	0.189
	High FAS	27 (2.5)		461 (42.1)		15 (1.4)	
2017/2018	Low FAS	67 (5.6)	**0.037**	493 (40.9)	0.369	33 (2.7)	**0.005**
	High FAS	27 (3.5)		299 (38.9)		7 (0.9)	

Note: FAS—family affluence scale, Abs.—absolute number and Diff.—difference; Bold font indicates the presence of statistical significance.
